# Supplementary material for: Empirical methods for controlling false positives and estimating confidence in ChIP-Seq peaks
Source: BMC Bioinformatics. 2008 Dec 5;9:523. doi: 10.1186/1471-2105-9-523 (PMC2628906; doi:10.1186/1471-2105-9-523)
Supplement: Additional File 1 — A variety of html documents from the USeq web site detailing the available applications, their best usage, output file type descriptions, command line menus, etc. [file 1471-2105-9-523-S1.zip › USeqUserGuides/JavaDoc.zip!/JavaDoc/bioroot/strain/StrainBase.html]

StrainBase


|  |  |  |
| --- | --- | --- |
| **Overview** \| **Package** \| **Class** \| **Use** \| **Tree** \| **Deprecated** \| **Index** \| **Help** | |  |
| **PREV CLASS**   **NEXT CLASS** | **FRAMES**    **NO FRAMES**     **All Classes** |  |
| SUMMARY: NESTED \| FIELD \| CONSTR \| METHOD | DETAIL: FIELD \| CONSTR \| METHOD |  |


---


## bioroot.strain Class StrainBase

```
java.lang.Object
  javax.servlet.GenericServlet
      javax.servlet.http.HttpServlet
          bioroot.strain.StrainBase
```

**All Implemented Interfaces:**: java.io.Serializable, javax.servlet.Servlet, javax.servlet.ServletConfig

---

``` public class StrainBase extends javax.servlet.http.HttpServlet ```

**Author:**
:   nix
    Contains methods for Strains

**See Also:**: Serialized Form

---

| **Field Summary** | |
| --- | --- |
| `static java.lang.String[]` | `searchHeadings`             Use to make a hash of column heading in which to search, name then the actual search column name. |
| `static java.lang.String[]` | `stringFeatures`             Use to make a default strain Feature[]. |


**Constructor Summary**

`StrainBase()`


| **Method Summary** | |
| --- | --- |
| `void` | `doGet(javax.servlet.http.HttpServletRequest request, javax.servlet.http.HttpServletResponse response)` |
| `void` | `doPost(javax.servlet.http.HttpServletRequest request, javax.servlet.http.HttpServletResponse response)` |
| `static java.lang.String[]` | `getSearchColumnNames()`             Returns a list of the search column names used in an SQL statment , ie name, 1/2 the searchHash. |
| `static java.util.LinkedHashMap` | `getSearchHash()` |
| `static java.lang.String[]` | `getSearchNames()` |
| `static java.lang.String` | `getStrainForm(StrainBean sb, DBUtil bioRoot, UserBean userBean)`             Returns a strain form filled with the values in the bean. |
| `static java.lang.String` | `getStrainReport(StrainBean sb, DBUtil bioRoot, UserBean userBean, boolean commonForm)`             Returns an strain report. |
| `static StrainBean[]` | `reverseStrainBeans(StrainBean[] beans)`             Reverses the order of an array of StrainBeans. |

**Methods inherited from class javax.servlet.http.HttpServlet**

`service`

**Methods inherited from class javax.servlet.GenericServlet**

`destroy, getInitParameter, getInitParameterNames, getServletConfig, getServletContext, getServletInfo, getServletName, init, init, log, log`

**Methods inherited from class java.lang.Object**

`equals, getClass, hashCode, notify, notifyAll, toString, wait, wait, wait`

**Field Detail**

### stringFeatures

```
public static java.lang.String[] stringFeatures
```

:   Use to make a default strain Feature[]. Trues must be on top.

---


### searchHeadings

```
public static final java.lang.String[] searchHeadings
```

:   Use to make a hash of column heading in which to search, name then the actual search column name.


**Constructor Detail**

### StrainBase

```
public StrainBase()
```


**Method Detail**

### getSearchHash

```
public static java.util.LinkedHashMap getSearchHash()
```

---


### getSearchNames

```
public static java.lang.String[] getSearchNames()
```

---


### getSearchColumnNames

```
public static java.lang.String[] getSearchColumnNames()
```

:   Returns a list of the search column names used in an SQL statment , ie name, 1/2 the searchHash.

---


### doGet

```
public void doGet(javax.servlet.http.HttpServletRequest request,
                  javax.servlet.http.HttpServletResponse response)
           throws javax.servlet.ServletException,
                  java.io.IOException
```

:   **Overrides:**: `doGet` in class `javax.servlet.http.HttpServlet`
:   **Throws:**: `javax.servlet.ServletException`: `java.io.IOException`

---


### doPost

```
public void doPost(javax.servlet.http.HttpServletRequest request,
                   javax.servlet.http.HttpServletResponse response)
            throws javax.servlet.ServletException,
                   java.io.IOException
```

:   **Overrides:**: `doPost` in class `javax.servlet.http.HttpServlet`
:   **Throws:**: `javax.servlet.ServletException`: `java.io.IOException`

---


### reverseStrainBeans

```
public static StrainBean[] reverseStrainBeans(StrainBean[] beans)
```

:   Reverses the order of an array of StrainBeans.

---


### getStrainReport

```
public static java.lang.String getStrainReport(StrainBean sb,
                                               DBUtil bioRoot,
                                               UserBean userBean,
                                               boolean commonForm)
```

:   Returns an strain report.

---


### getStrainForm

```
public static java.lang.String getStrainForm(StrainBean sb,
                                             DBUtil bioRoot,
                                             UserBean userBean)
```

:   Returns a strain form filled with the values in the bean. Must add a form entry. ie
    + "

    " and start and stop HTML stuff


---


|  |  |  |
| --- | --- | --- |
| **Overview** \| **Package** \| **Class** \| **Use** \| **Tree** \| **Deprecated** \| **Index** \| **Help** | |  |
| **PREV CLASS**   **NEXT CLASS** | **FRAMES**    **NO FRAMES**     **All Classes** |  |
| SUMMARY: NESTED \| FIELD \| CONSTR \| METHOD | DETAIL: FIELD \| CONSTR \| METHOD |  |


---
